# Quasi-Static Tests of Hybrid Adhesive Bonds Based on Biological Reinforcement in the Form of Eggshell Microparticles

**DOI:** 10.3390/polym12061391

**Published:** 2020-06-22

**Authors:** Viktor Kolář, Miroslav Müller, Rajesh Mishra, Anna Rudawska, Vladimír Šleger, Martin Tichý, Monika Hromasová, Petr Valášek

**Affiliations:** 1 Department of Material Science and Manufacturing Technology, Faculty of Engineering, Czech University of Life Sciences Prague, Kamycka 129, 165 00 Prague 6-Suchdol, Czech Republic; vkolar@tf.czu.cz (V.K.); muller@tf.czu.cz (M.M.); martintichy@tf.czu.cz (M.T.); valasekp@tf.czu.cz (P.V.); 2 Faculty of Mechanical Engineering, Lublin University of Technology, Nabystyczyka 36 Str., 20-618 Lublin, Poland; a.rudawska@pollub.pl; 3 Department of Mechanical Engineering, Faculty of Engineering, Czech University of Life Sciences Prague, Kamycka 129, 165 00 Prague 6-Suchdol, Czech Republic; sleger@tf.czu.cz; 4 Department of Electrical Engineering and Automation, Faculty of Engineering, Czech University of Life Sciences Prague, Kamycka 129, 165 00 Prague 6-Suchdol, Czech Republic; hromasova@tf.czu.cz

**Keywords:** cyclic loading, hybrid adhesive bond, mechanical properties, polymer composite, service life

## Abstract

The paper is focused on the research of the cyclic loading of hybrid adhesive bonds based on eggshell microparticles in polymer composite. The aim of the research was to characterize the behavior of hybrid adhesive bonds with composite adhesive layer in quasi-static tests. An epoxy resin was used as the matrix and microparticles of eggshells were used as the filler. The adhesive bonds were exposed to cyclic loading and their service life and mechanical properties were evaluated. Testing was performed by 1000 cycles at 5–30% (165–989 N) and 5–70% (165–2307 N) of the maximum load of the filler-free bond in the static test. The results of the research show the importance of cyclic loading on the service life and mechanical properties of adhesive bonds. Quasi-static tests demonstrated significant differences between measured intervals of cyclic loading. All adhesive bonds resisted 1000 cycles of the quasi-static test with an interval loading 5–30%. The number of completed quasi-static tests with the interval loading 5–70% was significantly lower. The filler positively influenced the service life of adhesive bonds at a higher amount of quasi-static tests, i.e., the safety of adhesive bonds increased. The filler had a positive effect on adhesive bonds ABF2, where the strength significantly increased up to 20.26% at the loading of 5–30% against adhesive bonds ABF0. A viscoelasticity characteristic (creep) of the adhesive layer occurred at higher values of loading, i.e., between loading 5–70%. The viscoelasticity behavior did not occur at lower values of loading, i.e., between loading 5–30%.

## 1. Introduction

The structural adhesive bonding process is one of the most important technologies for bonding various materials. In addition to their primary role in bonding materials, adhesive bonds can be also used in sealing, protecting or fastening. Adhesive bonds are widely used due to their properties in the automotive, agronomy, and aerospace industries, among others [1,2,3]. Many researchers are currently investigating the adhesive bonding technology [4,5,6]. The strength of adhesive bonds is the main factor in the structural adhesive bonding technology. The strength of adhesive bonds is influenced by many factors, such as wettability, adhesion, cohesion, adherend surface roughness, environment, size of filler and type of loading [7,8,9,10].

The current trend is to use adhesive bonds with an adhesive composite layer, where biological materials are used as a filler [11,12,13]. Biological materials can be used as substitutions of synthetic materials [14,15,16]. Some researchers have dealt with mechanical properties of polymeric composites with a hard filler from various shells [17,18,19,20,21,22,23]. This research deals with the utilization of the biological material as the adhesive bond filler based on eggshells in the microparticle form.

The eggshell makes up approx. 11% of the total weight of an egg. The food industry creates waste from eggshells, weighing about 250,000 tons, and the total cost for the utilization of the waste is about 14 million EUR [18,24]. The eggshell contains 95% calcium carbonate and the rest constitutes organic material, e.g., sulphate polysaccharides, type X collagen and many other proteins [25,26]. Eggshells are considered as a perspective additive into biodiesel due to a high temperature resistance, mechanical properties and easy recycling [27].

Eggshells are used in composite materials such as a source of calcium filler [28] and research has revealed that they have a significant influence in various products [26,29,30]. Eggshells, as biological fillers, can improve the mechanical properties, i.e., the tensile lap-shear strength, elongation at break, modulus of elasticity and thermal stability of adhesive bonds [29]. Müller et al. demonstrated that the filler in microparticle form can improve the mechanical properties of adhesive bonds, but the research was realized only for the static tensile test [31]. The cyclic loading test, which simulates a better and realistic condition of use of the adhesive bonds, is essential for the evaluation of service life.

Cyclic loading has an essential influence on the service life of adhesive bonds and an associated decrease of the mechanical properties. The decrease of mechanical properties can lead to a premature fatigue destruction of adhesive bonds, i.e., the adhesive bonds do not meet their functional requirement [11,32,33], which is undesirable for practical use. The cyclic fatigue of a material is the most destructive form of stress. It is an irreversible process which occurs at relatively lower forces [34]. The cyclic stress of adhesive bonds presents the most common cause of the degradation of these materials in practical use [35,36]. This undesirable phenomenon damages the integrity of adhesive bonds [37]. First, adhesive bonds are damaged, i.e., at the interface of the adhesive layer and bonded material. Second, damage occurs inside of the adhesive layer, i.e., the cohesive bonds are damaged. The fatigue destruction can occur already at low cyclic loading of adhesive bonds [11,32,33]. The strength of an adhesive bond is not only influenced by the added filler, but also by the distribution of the stress between elements of the filler and matrix. When the stress distribution between elements is more effective, it positively increases the adhesive bond strength, if the wettability of a filler and matrix is optimal [10,11,34,35,38,39]. 

The aim of the research was to characterize behavior of hybrid adhesive bonds with the biological filler, i.e., the eggshell microparticles, in the quasi-static tests. With various loading intensities and the establishment of the cyclic loading, there was a significant effect on the service life of the adhesive bonds based on the research results. The cyclic loading can be problematic for the safety and service life of adhesive bonds and therefore it is necessary to pay increased attention to this research.

## 2. Materials and Methods 

Hybrid adhesive bonds with the microparticle fillers from eggshells were the subject of this research. Adhesive bonds were exposed to cyclic loading and the service life with mechanical properties (tensile shear strength, elongation at break) were evaluated. The cyclic loading, with respect to the cyclic material fatigue, is the most destructive form of mechanical stress, where even a small force can cause delamination of an adhesive and can influence mechanical properties as well as the service life of adhesive bonds. These facts led to the following experiment. 

**Figure 1:**
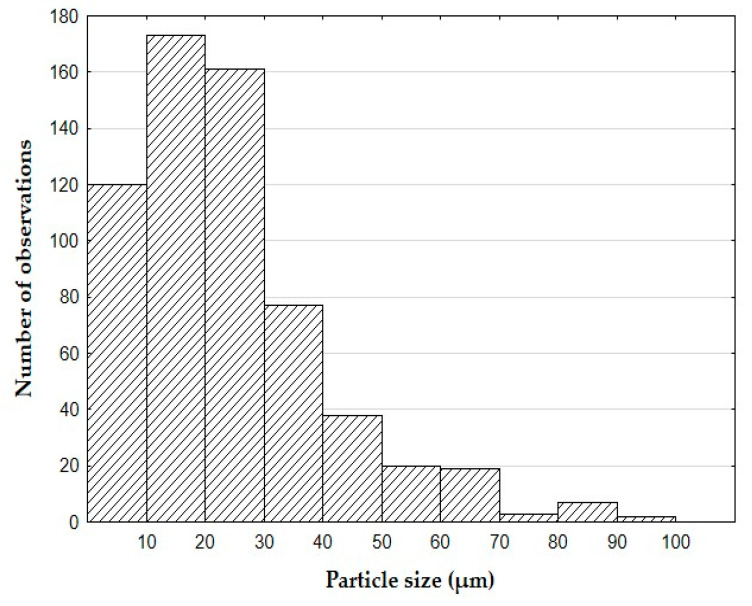
The histogram of particle size frequency of eggshell filler.

Eggshells for the filler were dried out in a laboratory chamber at temperature 105 °C for 24 hours. Dried eggshells were crushed to individual fractions on a vibrating sifter. The particle size was measured by the laser particle analyzer Horiba LA–960 VA (Horiba, Kyoto, Japan). The results of fraction size of the filler particles are shown in Table 1. The frequency histogram of the fraction size of the eggshell particles is presented in Figure 1. 

**Figure 2:**
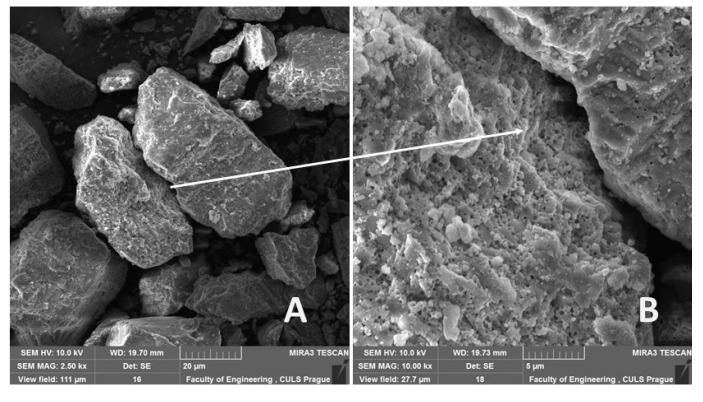
Microparticles of eggshell filler: (**A**): the filler shape and size (MAG 2.50 k×), (**B**): micro porosity on surface of the filler (MAG 10.00 k×).

The surface on the microparticle filler is porous which is evident from Figure 2B. Microparticles of the eggshell filler are characterized by a regular shape without sharp edges (Figure 2A).

The structural epoxy resin CHS-Epoxy 324 (Epoxy 1200) (Havel Composites CZ s. r. o., Svésedlice, Czech Republic) with hardener P11 was used as the matrix. The epoxy and hardener were mixed in the ratio 100:7 according the material supplier manual. This epoxy is suitable for preparation of a polymer composite to metal adhesive bonding.

**Figure 3:**
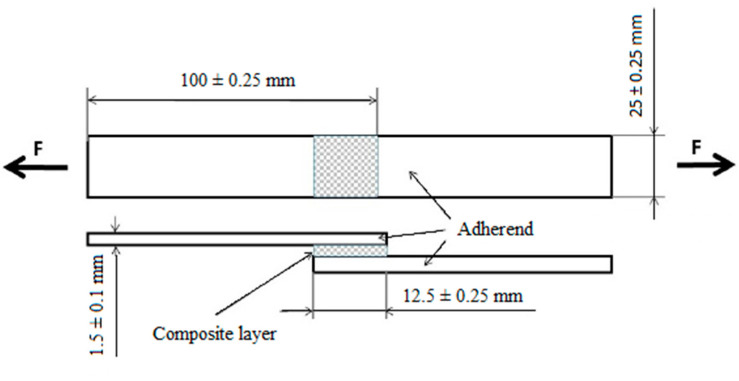
Adhesive bond according to ČSN EN 1465 [40].

The adherend was structural carbon steel S235J0 (Ferona a. s., Prague, Czech Republic) with size 100 mm × 25 mm × 1.5 mm according to the standard ČSN EN 1465 [40]. The adherend surface was mechanically treated in the blasting chamber by the abrasive Garnet MESH 80 and chemically cleaned by acetone. The roughness of the adherend surface was measured on Ra = 1.67 ± 0.16 μm and Rz = 9.98 ± 0.57 μm with the profilometer Mitutovo Surftest 301 (Mitutoyo Europe GmbH, Neuss, Germany). A limit wavelength of the cut-off was set as 0.8 mm. These adherend treatments were selected as the optimal from reported literature [39,41]. Adhesive bonds with the composite layer were made according to the standard ČSN EN 1465. The adhesive layer thickness was measured on 0.451 ± 0.011 µm by the Gwyddion program. Figure 3 shows the scheme of adhesive bonds with dimensions. Individual samples and characteristics of adhesive bonds are listed in Table 2. 

Mechanical properties were tested on the universal testing machine LABTest 5.50 ST with measuring unit AST KAF 50 kN and evaluation software Test and Motion (LABORTECH s. r. o., Opava, Czech Republic) under controlled laboratory temperature and humidity. The testing methods of mechanical properties in cyclic loading, i.e., shear tensile strength and elongation at break, were based on the reference value from static tensile test. The static tensile test was measured according to ČSN EN 1465 with adhesive bonds ABFE0 and ABFC0 at loading speed 0.6 mm×min^−1^ with 6 samples. This static reference value was 3296 ± 313 N (average maximal force from 6 samples) and corresponds to maximal force, i.e., the complete break of adhesive bond. The quasi-static test of the adhesive bonds was realized by 1000 loading cycles with test speed 0.6 mm×min^−1^ and two different loading values, i.e., lower loading interval 5–30% (165–989 N) from the static reference value and higher loading interval 5–70% (165–2307 N) from the reference value. When the 1000 cycles passed, adhesive bonds were statically loaded until the break by the static test with loading speed 0.6 mm×min^−1^. The time delay in loading interval was 0.5 s. The static tensile test was realized only in the case where the tested adhesive bond resisted all 1000 cycles, i.e., if the destruction occurred before 1000 cycles, the test was stopped. The tested series contained 6 samples.

The measured values were evaluated by the analysis of variance (ANOVA) F-test in the program STATISTICA. The statistical hypothesis was evaluated in significant level 0.05 between the adhesive bond without filler and samples with the filler. The statistical hypothesis H_0_ presents statistically nonsignificant differences between measured values (*p* > 0.05). The hypothesis H_1_ rejects hypothesis H_0_ and presents statistically significant differences between measured values (*p* <0.05).

The hybrid adhesive layer of adhesive bond, i.e., the interaction on filler–matrix interface was evaluated by an electron microscope MIRA 3 TESCAN GMX SE (Tescan Brno s. r. o., Brno, Czech Republic). The samples for microscopy were gold dusted by a Quorum Q150R ES device (Tescan Brno s. r. o., Brno, Czech Republic). 

## 3. Results and Discussion

**Figure 4:**
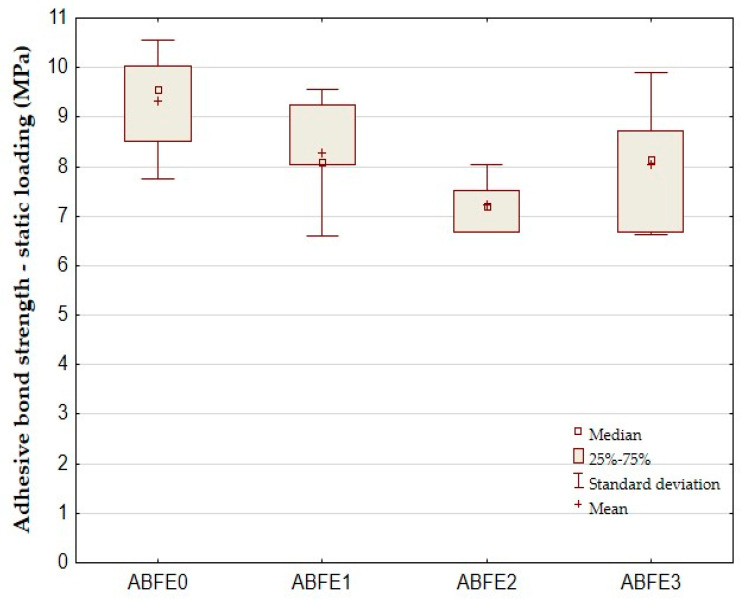
Static tensile test of adhesive bonds—shear tensile strength.

**Figure 5:**
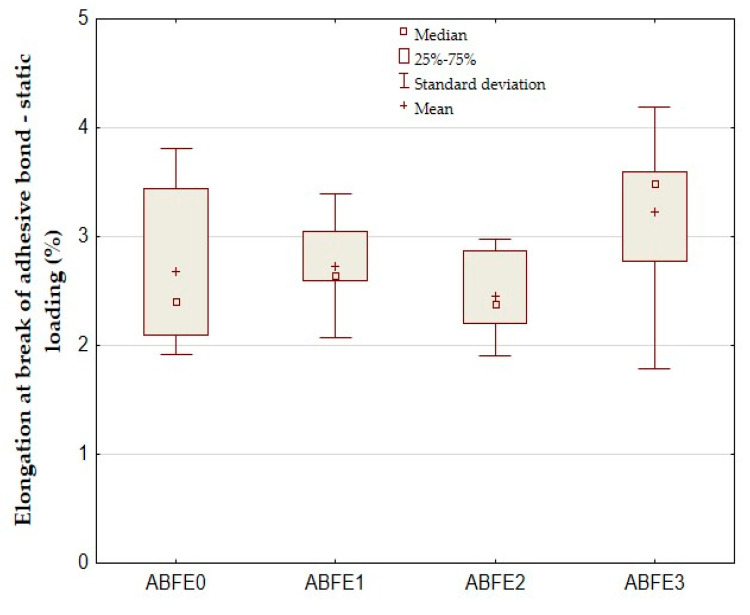
Static tensile test of adhesive bonds—elongation at break.

**Figure 6:**
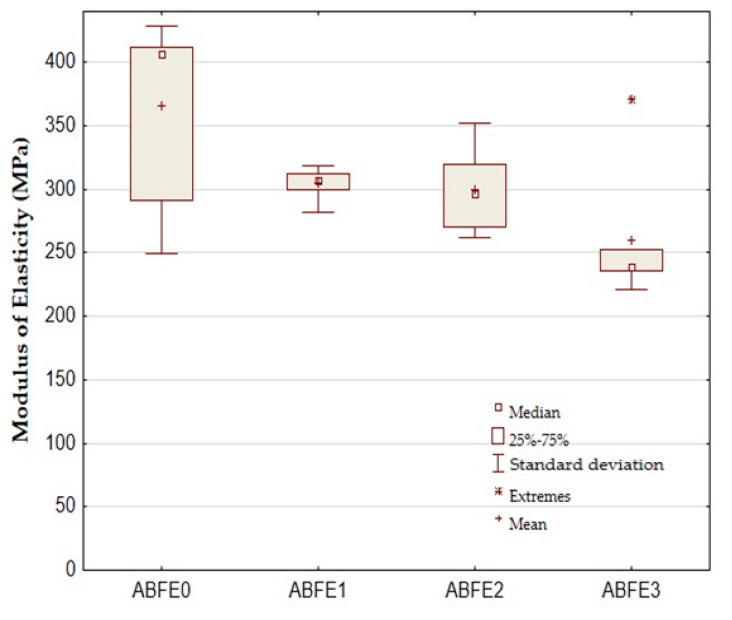
Static tensile test of adhesive bonds—modulus of elasticity.

The results of tested hybrid adhesive bonds in the statistic tensile test are evident in Figure 4, Figure 5 and Figure 6. Figure 4 presents the shear tensile strength, Figure 5 the elongation at break and Figure 6 the modulus of elasticity. Figure 4 shows the average shear tensile strength 9.33 ± 0.94 MPa of adhesive bonds ABFE0 in the static tensile test. The strength of adhesive bonds of ABFE1 was 8.27 ± 0.97 MPa. The strength decreased against ABFE0 up to 12.36%. The maximum decrease in strength occurred for adhesive bonds ABFE2 up to 22.62% at 7.22 ± 0.48 MPa. It follows that added eggshell microparticles negatively influence the adhesive bond strength during the static tensile test.

The statistical hypothesis confirms a difference between the adhesive bond ABFE0 (no filler) and adhesive bond ABFE2 at significance level 0.05. Hypothesis H_0_ was rejected, i.e., there is a significant difference between measured values. Parameter *p* = 0.0012, as seen in Table 3. As can be observed from the results, the filler negatively influenced the shear tensile strength of adhesive bonds.

Figure 5 shows a positive influence of adhesive bonds with eggshell fillers on the elongation at break. The average elongation at break of adhesive bonds ABFE0 was 2.68 ± 0.70%. The increase up to 1.87% at 2.73 ± 0.41% occurred for adhesive bonds ABFE1 against ABFE0. The maximum increase of elongation at break up to 20.15% occurred for adhesive bonds ABFE3 against ABFE0 at 3.22 ± 0.76%. 

The statistical hypothesis did not confirm a difference between the adhesive bond without the filler and samples with the filler at significance level 0.05, as seen in Table 3. The hypothesis H_0_ was admitted, i.e., there is no significant difference between measured values. It is apparent that the filler did not have significant influence on the change in the elongation at break.

The modules of elasticity of adhesive bonds in the tensile shear test are shown in Figure 6. A decrease of the modules of elasticity after adding the filler is evident from the results. The average value of the modulus of elasticity for adhesive bond ABFE0 was 365.38 ± 68.81 MPa. The filler decreased the modulus of elasticity value against ABFE0 from 16.67% to 20.93%. The worst results were achieved for the adhesive bond ABFE3, as shown in Figure 5. The static tests of the modulus of elasticity showed a statistically significant difference between the adhesive bond ABFE0 (no filler) and ABFE3 at significance level 0.05, as seen in Table 3. Hypothesis H_0_ was rejected. The filler had significant influence on the modulus of elasticity, i.e., p = 0.0219.

**Figure 7:**
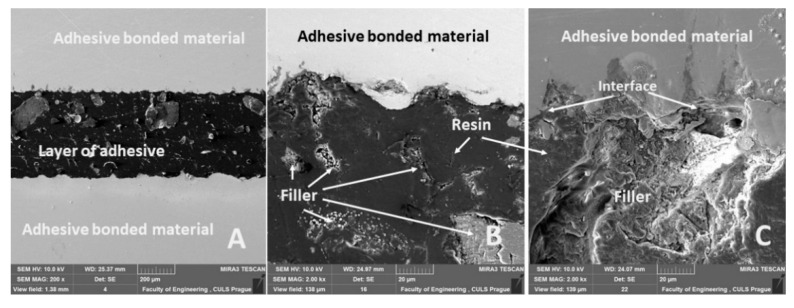
Adhesive bond ABFE2 cross-section: (**A**): total view of adhesive bond (MAG 200 ×), (**B**) filler distribution in adhesive layer and interface of resin, filler and bonded material (MAG 2.00 k×), (**C**): detailed view of interface of adhesive layer and bonded material (MAG 2.00 k×).

The adhesive bond cross-section with the eggshell microparticles is shown in Figure 7, which presents the integrity of adhesive bonds and the interface of the filler and resin. Figure 7A presents the cross-section of the whole adhesive bond, i.e., the adhesive layer (resin and filler), bonded material (structural carbon steel S235J0) and the interface of adherend and adhesive layer. Figure 7B,C show a detailed view of the distribution and shape of the filler in the adhesive bond and their interface. A good adhesion interface between the adhesive, filler and adherend is evident from Figure 7. This factor is the basic success of an adhesive bond [41].

**Figure 8:**
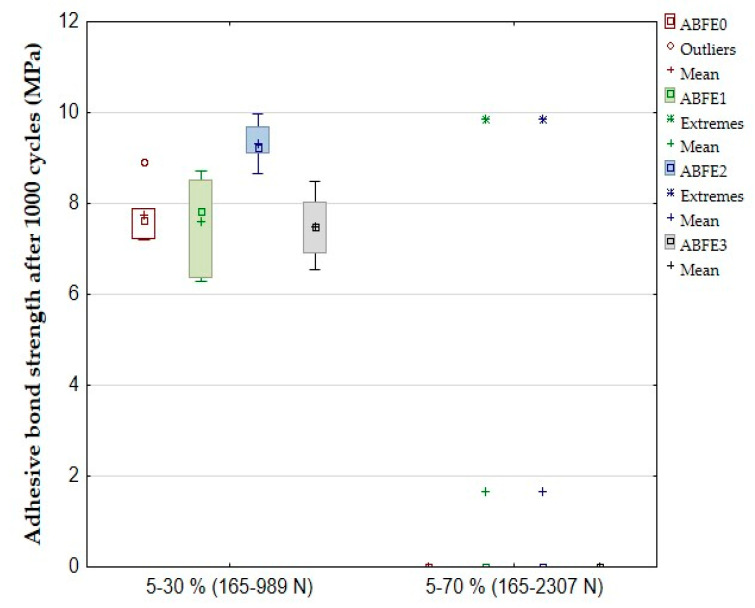
Quasi-static test of adhesive bonds—shear tensile strength after 1000 cycles.

**Figure 9:**
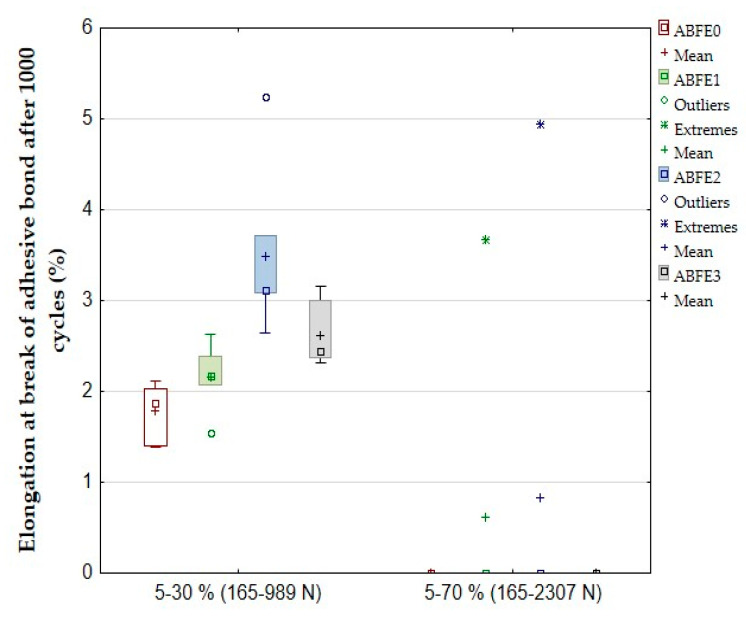
Quasi-static test of adhesive bonds—elongation at break after 1000 cycles.

The results of the quasi-static test after 1000 cycles are evident from Table 4 and Figure 8 and Figure 9. Table 4 shows a difference in the number of adhesive bonds, which resisted the cyclic loading with above 1000 cycles. All adhesive bonds (6/6) resisted the cyclic loading between 5–30% with above 1000 cycles. The adhesive bonds did not resist the cyclic loading between 5–70% with 1000 cycles. The results of the quasi-static test 5–70% (165–2307 N) in Table 4 show that a viscoelasticity behavior (the relative deformation) occurred inside of the adhesive layer between first and thousandth cycle for adhesive bonds ABFE0 up to 0.25%, ABFE1 up to 0.17%, ABFE2 up to 0.19% and ABFE3 up to 0.14%. The viscoelasticity behavior, i.e., the creep, causes a deformation of adhesive bonds [11]. The results show that the creep negatively influenced the resistance of the adhesive bonds in the quasi-static test with the higher loading (5–70%). The results in Table 4 show that repeated cyclic loading of the adhesive bonds with higher values of loading force, approaching the maximum loading force during the static test, could lead to the premature failure of the adhesive bond with a relatively small number of cycles [11,37].

The viscoelastic behavior inside of adhesive layer at quasi-static test 5–30% (165–989 N) was not proved. The results in Table 4 show a change of the relative deformation between the first and thousandth cycle for adhesive bonds ABFE0 up to 0.01%, ABFE1 up to 0.02%, ABFE2 up to 0.02% and ABFE3 up to 0.05%. The relative deformation between the first and thousandth cycle was 0.025 ± 0.005%. The similar results were demonstrated for adhesive bonds with cotton microparticles and short fibers [11]. This result is very important for practical use because the filler can increase the service life and safety of the adhesive bonds under cyclic stress.

Figure 8 shows the average shear strength 7.75 ± 0.58 MPa of adhesive bond ABF0 during the quasi-static test between 5–30%. The added filler in adhesive bond ABFE2 positively increased the strength up to 20.26% at 9.32 ± 0.42 MPa. The adhesive bonds ABFE1 reported a decrease of the strength up to 3.1% and adhesive bonds ABFE3 up to 3.42%. The average strength of adhesive bond ABFE0 was 6.56 ± 0.39 MPa at loading between 5–70%. A slight increase of the strength up to 10.98% at 7.28 ± 1.18 MPa occurred for adhesive bond ABFE1.

The filler significantly influenced the elongation at break between loading 5–30%, as can be seen in Figure 9. The elongation at break improved for all adhesive bonds (ABFE1, ABFE2, ABFE3) against ABFE0. The highest increase in elongation at break up to 96.06% occurred for adhesive bonds ABFE2. The elongation at break improved up to 47.19% for ABFE3 and up to 21.35% for ABFE1. The elongation at break cannot be considered significant because the adhesive was destroyed before 1000 cycles, as shown in Table 4.

The results from statistical testing are shown in Table 5. It is evident that the statistical tests after cyclic loading 5–30% (165–989 N) proved a significant difference in the shear tensile strength and elongation at break between the adhesive bond without filler and samples with the filler at significance level 0.05. The results of the quasi-static test 5–70% (165–2307 N) cannot be considered significant because the adhesives were destroyed before 1000 cycles.

The results of the research corresponded with a statement—that adding a filler into a matrix can lead to a negative effect, i.e., decrease of the cyclic loading of adhesive bonds [42,43].

**Figure 10:**
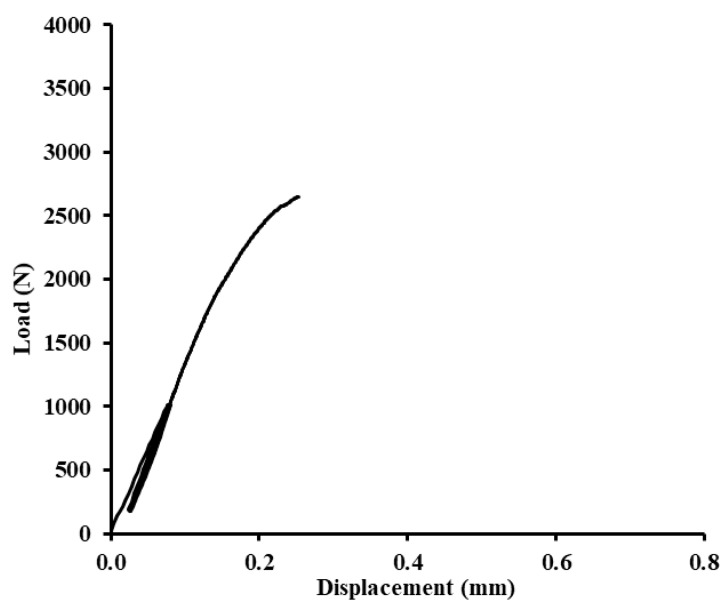
Quasi-static graph of adhesive bond ABFE0 with loading 5–30% (165–989 N)—1000 cycles.

**Figure 11:**
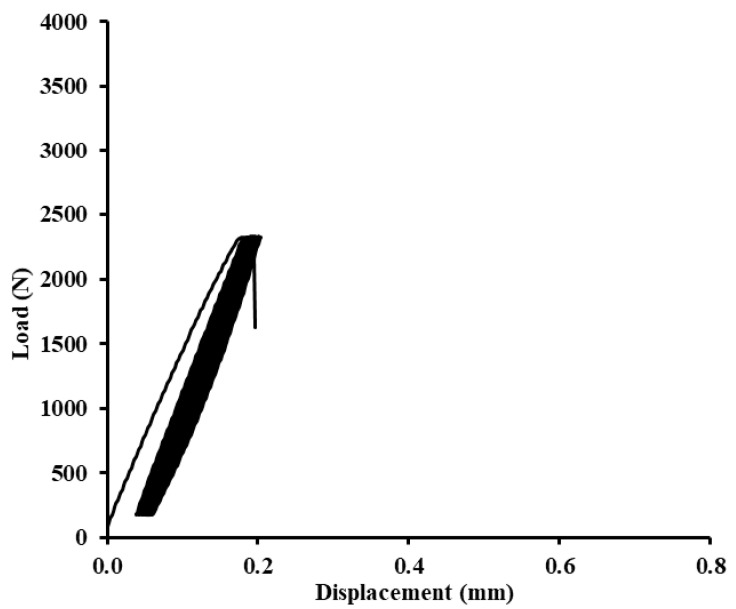
Quasi-static graph of adhesive bond ABFE0 with loading 5–70% (165–2307 N)—256 cycles.

**Figure 12:**
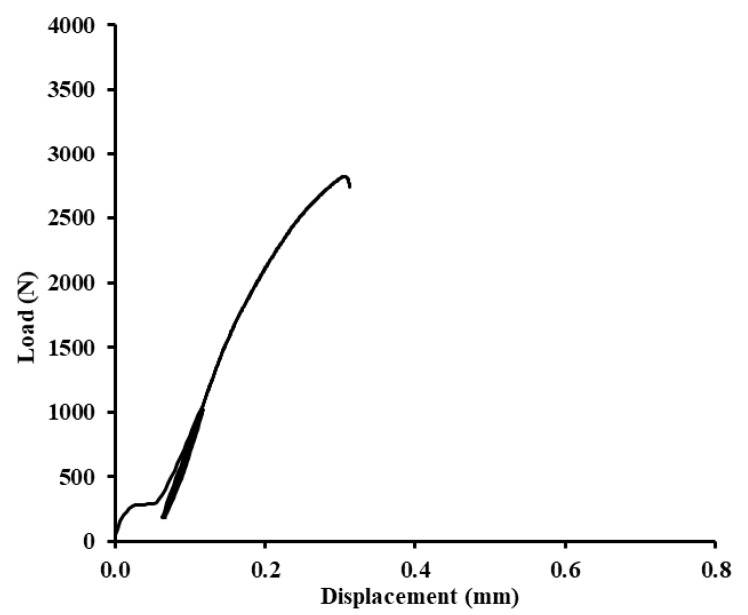
Quasi-static graph of adhesive bond ABFE1 with loading 5–30% (165–989 N)—1000 cycles.

**Figure 13:**
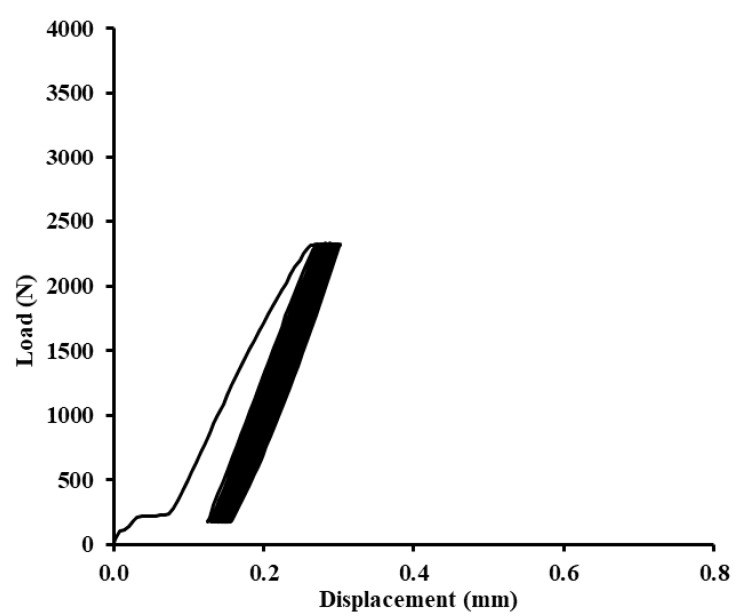
Quasi-static graph of adhesive bond ABFE1 with loading 5–70% (165–2307 N)—734 cycles.

**Figure 14:**
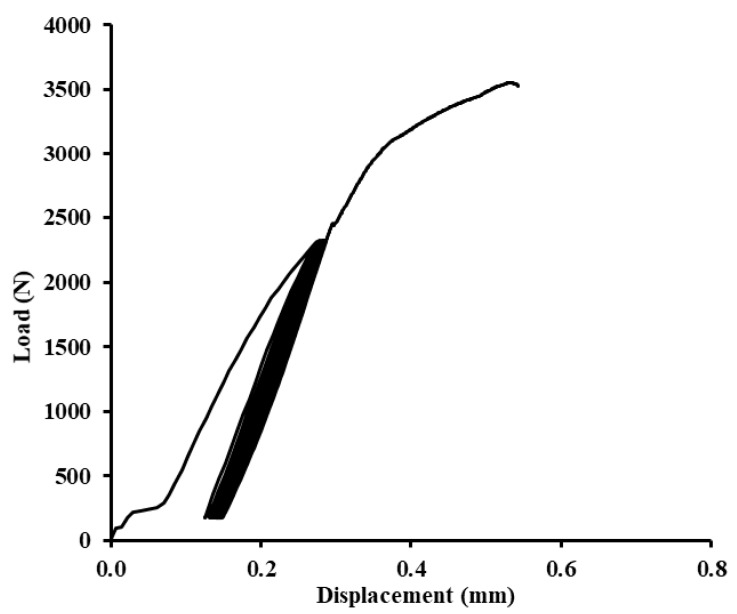
Quasi-static graph of adhesive bond ABFE1 with loading 5–70% (165–2307 N)—1000 cycles.

**Figure 15:**
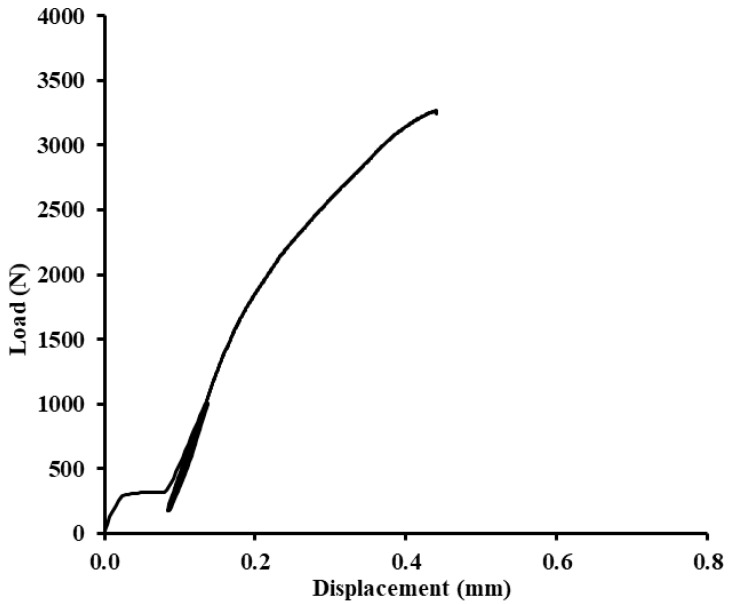
Quasi-static graph of adhesive bond ABFE2 with loading 5–30% (165–989 N)—1000 cycles.

**Figure 16:**
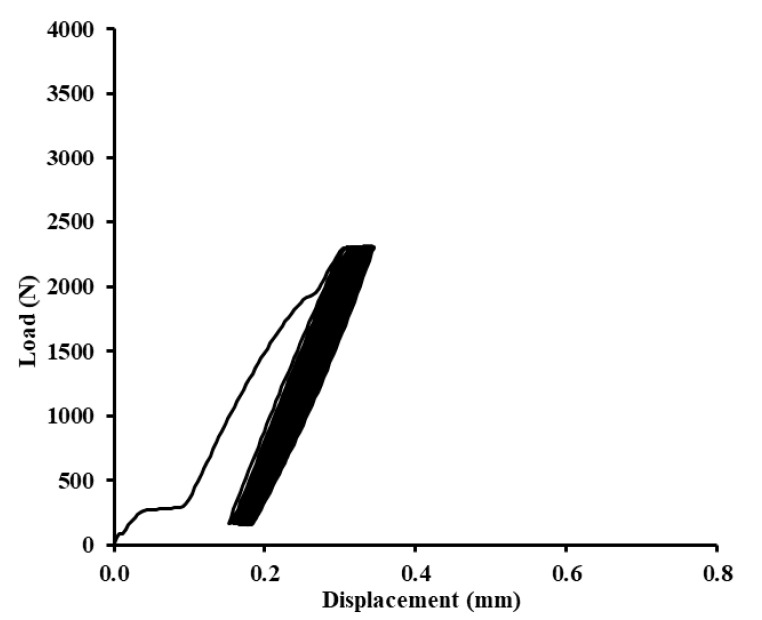
Quasi-static graph of adhesive bond ABFE2 with loading 5–70% (165–2307 N)—267 cycles.

**Figure 17:**
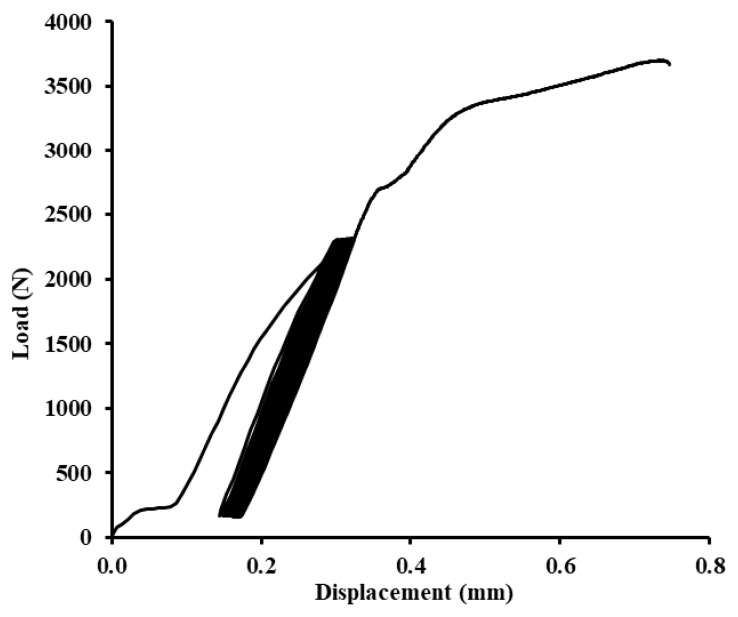
Quasi-static graph of adhesive bond ABFE2 with loading 5–70% (165–2307 N)—1000 cycles.

Examples of quasi-static graphs are shown in Figure 10, Figure 11, Figure 12, Figure 13, Figure 14, Figure 15, Figure 16 and Figure 17. Figure 10 presents the quasi-static graph of the adhesive bond without filler, ABFE0 with loading 5–30% (165–989 N) and Figure 11 presents loading 5–70% (165–2307 N) of the static reference value. In Figure 10, it is evident that all 1000 cycles were finished and the static tensile test was completed (without removing tested sample from testing machine between last cycle and final deformation). In Figure 11, it is evident that the adhesive bond with a higher loading value, i.e., 5–70% (165–2307 N), resisted only 256 cycles. Adhesive bond failure occurred, and the following static test was not realized.

In Figure 12, Figure 13 and Figure 14, examples of quasi-static graphs of selected hybrid adhesive bonds with the composite layer of adhesive are presented, ABFE1 with loading 5–30% (165–989 N) and 5–70% (165–2307 N). Figure 12 presents the quasi-static graph of the hybrid adhesive bond ABFE1 at 5–30% after 1000 cycles. Figure 13 presents the adhesive bond ABFE1 at 5–70% after 734 cycles and Figure 14 presents the adhesive bond ABFE1 at 5–70% after 1000 cycles. 

In Figure 15, Figure 16 and Figure 17, examples of quasi-static graphs of selected hybrid adhesive bonds with composite layer of adhesive are presented, ABFE2 with loading 5–30% (165–989 N) and 5–70% (165–2307 N). Figure 15 presents the quasi-static graph of the hybrid adhesive bond ABFE2 at 5–30% after 1000 cycles. Figure 16 presents the adhesive bond ABFE2 at 5–70% after 267 cycles and Figure 17 presents the adhesive bond ABFE2 at 5–70% after 1000 cycles.

**Figure 18:**
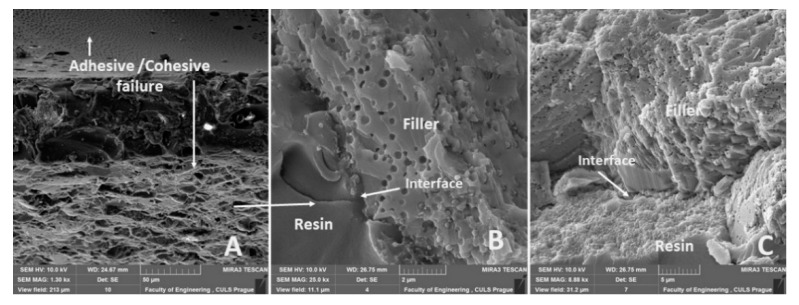
SEM images of fracture surface ABFE2: (**A**): adhesive/cohesive fracture surface of adhesive bond (MAG 1.30 k×, (**B**): detailed on adhesive/cohesive fracture surface and interface resin and filler and detailed view on filler surface (MAG 25.00 k×), (**C**): Cohesive fracture filler and detailed view on filler surface (MAG 8.88 k×).

Figure 18A shows the fracture surface inside of the adhesive layer, i.e., adhesive/cohesive failure type of the adhesive bond. The fracture surface of the adhesive bond was changed by adding the filler. The adhesive bonds without filler had an adhesive fracture surface. The adhesive bonds with the filler had an adhesively cohesion fracture surface from 80% (Figure 18A). Figure 18B,C present a good adhesive strength, i.e., a good interaction between matrix and eggshells microparticles. The surface of eggshell microparticles, which indicates porosity, is evident from Figure 18B,C. The porosity is used for good wettability of the matrix and filler. It leads to an adhesive surface enlargement (Figure 18C). From Figure 18B,C, it is evident that the adhesive moistens the micropores of the eggshell filler. Due to this phenomenon and good wettability, the functional surface for the filler and resin interaction is increased.

## 4. Conclusions

This paper deals with the mechanical properties of hybrid adhesive bonds with a biological filler from eggshells in the microparticle form. Adhesive bonds were tested by the quasi-static test and the adhesive bond strength and elongation at break were evaluated. Important conclusions are listed in following points:

- the static tensile test of adhesive bonds showed a decrease of the tensile strength of all adhesive bonds, i.e., ABF1, ABF2, ABF3 compared to ABF0. The statistical testing confirmed a statistically significant difference between the measured data (*p* < 0.05). The filler had a positive effect on the elongation at break for adhesive bonds ABF1 and ABF3, but statistical testing did not confirm a statistically significant difference between the measured values (*p* > 0.05),
- in the quasi-static tests between 5–30%, all adhesive bonds resisted the upper limit of 1000 cycles. Most of the adhesive bonds failed between 5–70% prematurely. It was confirmed that even a small number of cycles leads to the premature failure of adhesive bonds,
- the filler had a positive effect on the ABF2 adhesive bond, where there was a significant increase in the strength compared to ABF0 by 20.26% at the load between 5–30%. The strength of the adhesive bonds ABF1 and ABF3 decreased compared to ABF0 with an average up to 3.3%. For the elongation at break, the filler had a positive effect on all adhesive bonds between the load 5–30%. The most significant improvement was for ABF2 up to 96.06% compared to ABF0. The service life and safety of adhesive bonds increased. The statistical analysis showed significant differences between the measured values (*p* < 0.05) except for the strength of the ABF1 adhesive bonds (*p* > 0.05),
- SEM analysis showed a good wettability between the filler and matrix. SEM analysis showed the potential of the filler, especially in the area of the porosity of the micro surface that is wetted by the resin. The cross-section of the adhesive bond demonstrated a good integrity of all three essential layers presenting the adhesive bonds, i.e., the adhesive layer, the adhesion and cohesion layer.

## Figures and Tables

**Table 1:** Fraction size of the filler particles.

Arithmetic Mean (µm)	Mode (µm)	Median (µm)
28.80 ± 15.48	19.60	23.265

**Table 2:** Samples and characteristic of adhesive bonds with eggshells filler.

Sample	Characteristic
ABFE0	Adhesive bond without filler
ABFE1	Adhesive bond with filler (mass ratio 10:1)
ABFE2	Adhesive bond with filler (mass ratio 10:2)
ABFE3	Adhesive bond with filler (mass ratio 10:3)

**Table 3:** Statistical testing of adhesive bonds—adhesive bond shear strength, elongation at break and modulus of elasticity.

Testing of Adhesive Bonds under Shear Tensile Stress	ABFE0	ABFE1	ABFE2	ABFE3
Adhesive bond strength (MPa)	0.0000	0.1097	0.0012	0.0820
Elongation at break (%)	0.0000	0.8839	0.5401	0.2665
Modulus of Elasticity (MPa)	0.0000	0.1086	0.0779	0.0219

**Table 4:** Results of quasi-static test of adhesive bonds.

Adhesive Bond	Quasi-Static Test	Number of Finished Tests	Relative Deformation 1st Cycle (%)	Relative Deformation 1000th Cycle (%)
ABFE0	5%–30% 165 N–989 N	6/6	0.14 ± 0.06	0.15 ± 0.06
ABFE1		6/6	0.48 ± 0.04	0.50 ± 0.05
ABFE2		6/6	0.60 ± 0.05	0.62 ± 0.05
ABFE3		6/6	0.77 ± 0.07	0.82 ± 0.06
ABFE0	5%–70% 165 N–2307 N	0/6	0.35 ± 0.07	0.50 ± 0.09
ABFE1		1/6	0.87 ± 0.07	1.04 ± 0.10
ABFE2		1/6	1.04 ± 0.09	1.23 ± 0.06
ABFE3		0/6	1.16 ± 0.04	1.30 ± 0.09

**Table 5:** Statistical testing of adhesive bonds—adhesive bond shear strength and elongation at break after cyclic loading 5–30% (165–989 N).

Testing of Adhesive Bonds under Shear Tensile Stress	ABFE0	ABFE1	ABFE2	ABFE3
Adhesive bond strength (MPa)	0.0000	0.0058	0.0009	0.0000
Elongation at break (%)	0.0000	0.0333	0.0148	0.0000
